# Comparing the Outcomes of Cardiogenic Shock After Myocardial Infarction in Women Across Different Age Groups

**DOI:** 10.7759/cureus.85298

**Published:** 2025-06-03

**Authors:** Nformbuh Asangmbeng, Frank H Annie, Kerry Drabish, Sangeeta Mandapaka

**Affiliations:** 1 Department of Cardiology, Charleston Area Medical Center, Charleston, USA

**Keywords:** cardiogenic shock, cardiogenic shock-related mortality, female patients, myocardial infarction, postmyocardial

## Abstract

Background: Cardiogenic shock (CS) complicating myocardial infarction (MI) is associated with high morbidity and mortality, with age- and sex-specific differences in presentation and outcomes. Female patients, often underrepresented in cardiovascular research, exhibit distinct clinical profiles and treatment patterns. However, age-stratified outcomes among women with post-MI CS remain insufficiently described.

Aim: This study aimed to compare the outcomes of post-MI CS between female patients in two distinct age groups: 45-55 and 56-75 years.

Methods: We conducted a retrospective cohort study using the TriNetX Research Network, including deidentified electronic health records from 102 healthcare organizations between January 1, 2017, and January 1, 2023. Female patients aged 45-55 or 56-75 years with both acute MI (International Classification of Diseases, 10th Revision, ICD-10: I21) and CS (ICD-10: R57.0) were identified. Two cohorts were generated and matched 1:1 using propensity scores for demographics and comorbidities, resulting in 1,100 patients per group. Outcomes assessed included all-cause mortality, major adverse cardiac events (MACE), arrhythmias, atrial fibrillation (Afib), cardiac device procedures/complications, and emergency readmissions over a three-year follow-up. Outcomes were compared using Kaplan-Meier analysis and Cox proportional hazards models.

Results: Older women (56-75 years) had significantly higher three-year mortality compared to the younger cohort (41.2% vs. 36.2%, p = 0.016), as well as increased incidence of Afib (26.5% vs. 22.7%, p = 0.038). Rates of MACE and other arrhythmias were similar between the groups. Emergency readmissions were more common in younger women (33.7% vs. 29.3%, p = 0.025), while device-related procedures showed low event rates and group-specific trends. Absolute differences in outcomes were modest.

Conclusions: Age-related differences exist among female patients with post-MI CS. Older women face higher mortality and Afib risk, while younger women experience more frequent emergency readmissions. These findings highlight the need for age-specific postdischarge strategies, but must be interpreted cautiously given the study’s retrospective design, reliance on administrative coding, and lack of granular clinical data. Further prospective research is needed to elucidate the mechanisms and inform tailored interventions for women with CS following MI.

## Introduction

Cardiogenic shock (CS) is a severe, life-threatening complication of acute myocardial infarction (MI), associated with high morbidity and mortality rates despite advances in contemporary care [1-3]. Up to 40%-80% of CS cases are attributed to acute MI, and outcomes remain poor, particularly in vulnerable populations [2,3].

Sex and age differences in cardiovascular disease presentation and outcomes have been increasingly recognized. Female patients with MI and CS often present with a distinct risk factor profile, including a higher prevalence of hypertension, diabetes, and chronic pulmonary disease, and are more likely to receive nonprocedural management compared to men [4-6]. Multiple studies have shown that, after adjustment for confounders, women experience higher mortality from CS than men [1,5,6]. However, women have traditionally been underrepresented in clinical trials and large cardiovascular registries, limiting the ability to generalize findings and tailor interventions specifically for women [5,7].

The impact of age on outcomes in post-MI CS is also complex. Prior research suggests that sex differences in CS outcomes may vary by age group, with female patients in middle and older age groups experiencing greater risk compared to male patients, but data remain mixed and limited in granularity [7]. Notably, previous studies have focused primarily on comparing outcomes between sexes or have grouped female patients without age stratification, making it difficult to discern whether risk factors and outcomes differ between younger and older women with CS [6,7]. Understanding age-specific patterns is essential for developing targeted interventions and improving clinical management for female patients.

To address these gaps, we conducted a large, multicenter retrospective cohort study to compare outcomes of post-MI CS between female patients in two clinically relevant age groups: 45-55 years (encompassing perimenopausal and early postmenopausal women) and 56-75 years (older postmenopausal women). These age categories were chosen based on prior literature, highlighting changes in cardiovascular risk and outcomes across the menopausal transition and the need to ensure adequate sample sizes for robust statistical analysis [7]. Focusing solely on women allows for a more detailed characterization of risk and outcome differences within this understudied population.

The primary aim of this study is to evaluate whether age-related differences exist in mortality, major adverse cardiac events (MACEs), arrhythmias, atrial fibrillation (Afib), cardiac device utilization, and emergency readmissions among women with post-MI CS. By clarifying these patterns, we hope to inform age-appropriate strategies for post-MI CS management in women and highlight areas in need of further research.

## Materials and methods

We conducted a retrospective cohort study using the TriNetX Research Network (TriNetX, Cambridge, MA), a federated electronic health record (EHR) platform that aggregates deidentified patient-level data from 102 healthcare organizations across North America. The database includes standardized information on demographics, diagnoses, procedures, medications, laboratory results, and clinical encounters. The TriNetX platform performs regular quality control and mapping to ensure data consistency. Institutional Review Board approval was obtained (IRB protocol 25-1180); all data were deidentified, and the study was deemed nonhuman subject research. We identified female patients aged 45-75 years with both acute MI and CS between January 1, 2017, and January 1, 2023. MI was defined by the International Classification of Diseases, 10th Revision (ICD-10) code I21; CS was defined by ICD-10 code R57.0. The index event was the first hospitalization meeting both criteria within the study period.

Patients were stratified into two age cohorts at the time of the index event: 45-55 years (peri- and early postmenopausal) and 56-75 years (older postmenopausal). These age ranges were selected based on prior literature emphasizing cardiovascular risk transitions across the menopausal period and to ensure adequate power for comparative analyses. Patients younger than 45 or older than 75 were excluded to limit heterogeneity and due to small sample sizes in these groups. We excluded patients with incomplete demographic data, prior MI within six months before the index admission, missing outcome data, or lacking follow-up after the index event. Extracted variables included age, race/ethnicity, body weight, and comorbidities (hypertension, diabetes mellitus, chronic obstructive pulmonary disease, COPD, heart failure, and atherosclerotic disease). COPD was included due to its established association with adverse cardiovascular outcomes and its higher prevalence in female cardiac patients. Primary outcomes were all-cause mortality, MACE (defined as the composite of recurrent MI, stroke, acute ischemic heart disease, and heart failure), arrhythmias, Afib, cardiac device utilization/complications, and emergency readmissions within three years postindex event. Emergency readmissions were defined as any unplanned hospitalization via the emergency department after discharge from the index admission, regardless of cause, as per TriNetX standard definitions. Cardiac devices included pacemakers, cardiac resynchronization therapy (CRT) devices, implantable loop recorders, intra-aortic balloon pumps (IABP), left ventricular assist devices (LVADs)/right ventricular assist devices (RVADs)/total artificial heart (TAH), and atrial septal defect (ASD)/ventricular septal defect occluders. Device-related complications were included in the device outcome.

TriNetX employs internal mapping and validation routines; however, clinical diagnoses and procedures are based on ICD-10 codes, and chart-level validation was not feasible. The limitations of EHR coding and potential misclassification are acknowledged. To minimize confounding, 1:1 propensity score matching (PSM) was performed between the two age groups using a nearest-neighbor algorithm with a caliper width of 0.2 standard deviations (SDs). Matching variables included race/ethnicity, comorbidities (hypertension, diabetes, COPD, heart failure, and atherosclerotic disease), and body weight. Standardized mean differences (SMDs) <0.1 were considered indicative of adequate balance. Patients with missing data for covariates used in PSM were excluded; no data imputation was performed.

Continuous variables are presented as mean ± SD and compared using Student’s t-tests. Categorical variables are shown as counts and percentages, compared using chi-square or Fisher’s exact tests as appropriate. Time-to-event outcomes were analyzed using Kaplan-Meier survival analysis, with group differences assessed by the log-rank test. Hazard ratios (HRs) and 95% confidence intervals (CIs) were estimated using Cox proportional hazards regression models. For rare events and zero counts, Fisher’s exact test was used, and Cramér’s V was not reported for those comparisons. No formal adjustment for multiple comparisons was performed; the risk of type I error is acknowledged given the exploratory nature of the study. All statistical analyses were performed within the TriNetX platform.

## Results

Following the application of inclusion and exclusion criteria, a total of 2,200 female patients with both acute MI and CS were identified from 102 healthcare organizations in the TriNetX Network. After 1:1 PSM, 1,100 patients were retained in each age group: 45-55 and 56-75 years. Baseline demographic and clinical characteristics were well balanced between the cohorts, with SMDs <0.1 for all matched variables, including race, body weight, and comorbidities such as hypertension, diabetes, COPD, heart failure, and atherosclerotic disease. Mean follow-up was 565 days in the 45-55 cohort and 508 days in the 56-75 cohort. Three-year all-cause mortality was significantly lower in the 45-55 age group compared to the 56-75 group (36.2% vs. 41.2%; absolute risk difference: -5.0%, 95% CI: -9.5% to -1.9%; p = 0.016; risk ratio: 0.88, 95% CI: 0.79-0.98). Kaplan-Meier analysis demonstrated improved survival in the younger cohort (log-rank p = 0.001; HR: 0.82, 95% CI: 0.71-0.91). MACE occurred at a similar high rate in both groups (85.2% vs. 85.1%, p = 0.952; risk ratio: 1.00, 95% CI: 0.92-1.08; HR: 0.95, 95% CI: 0.87-1.04), with no significant difference in time-to-event curves. Rates of any arrhythmia were comparable between the groups (29.3% vs. 30.1%, p = 0.674; HR: 0.91, 95% CI: 0.80-1.14). Afib occurred more frequently in the older cohort (26.5% vs. 22.7%, p = 0.038; absolute risk difference: -3.8%; HR: 0.78, 95% CI: 0.61-0.84). Kaplan-Meier analysis showed a higher freedom from Afib in younger women (70.4% vs. 63.6% at three years, log-rank p < 0.001). The 45-55 group experienced more emergency readmissions compared to the 56-75 group (33.7% vs. 29.3%, p = 0.025; risk ratio: 1.15, 95% CI: 1.02-1.30; HR: 1.08, 95% CI: 0.97-1.21), despite a longer median time to first readmission (858 vs. 1,051 days).

All-cause readmissions included both cardiac and noncardiac events due to data constraints. Cardiac device utilization (composite outcome) was more frequent in the younger cohort but not statistically significant (6.3% vs. 4.8%, p = 0.136; HR: 1.22, 95% CI: 0.85-1.74). Use of IABP and paravalvular leak repair was higher in the younger group (both 0.9% vs. 0.0%, p = 0.002). ASD occluders were more often used in younger female patients (3.1% vs. 1.5%, p = 0.016; HR: 1.87, 95% CI: 1.05-3.35). Other procedures, including pacemaker implantation, CRT, LVAD/RVAD/TAH, mitral valve repair, and coronary stenting, showed no significant group differences. Due to the rarity of these events, effect size estimates should be interpreted with caution, and no Cramér’s V was reported for Fisher’s exact test comparisons. All comparisons are reported for matched cohorts. Statistical tests used for each outcome are detailed in Tables 1, 2. For outcomes with zero events in one group, Fisher’s exact test was applied. No imputation was performed for missing data; patients with incomplete covariate or outcome data were excluded prior to matching.

**Table 1:** Differences in outcomes between cohorts showing percentages for each age group, the combined total, and corresponding statistics (rank difference, risk ratio, hazard ratio, and p value)

Outcome	45-55 years (n = 1,100)	56-75 years (n = 1,100)	p value	Risk ratio	Risk difference (95% CI)	Hazard ratio
Mortality	398 (36.20%)	454 (41.20%)	0.016	0.88	-5%	0.82
Major adverse cardiac events	937 (85.20%)	936 (85.10%)	0.952	1	0.10%	0.95
Arrhythmias	322 (29.30%)	331 (30.10%)	0.674	0.97	-0.80%	0.91
Atrial fibrillation	250 (22.70%)	292 (26.50%)	0.038	0.86	-3.80%	0.78
Cardiac devices	69 (6.30%)	53 (4.80%)	0.136	1.3	1.50%	1.22
Readmissions	371 (33.70%)	322 (29.30%)	0.025	1.15	4.40%	1.08
Transcatheter aortic valve replacement	10 (0.90%)	10 (0.90%)	1	1	0%	0.79
Pacemakers	113 (10.20%)	99 (9%)	0.347	1.13	1.20%	1.06
Cardiac resynchronization therapy	88 (8%)	105 (9.50%)	0.227	0.85	-1.50%	0.77
Loop recorder	10 (0.90%)	10 (0.90%)	1	1	0%	0.87
Left ventricular assistive device/right ventricular assistive device/total artificial heart	24 (2.20%)	13 (1.20%)	0.068	1.85	1%	1.75
Intra-aortic balloon pump	10 (0.90%)	0 (0%)	0.002	-	0.90%	-
Mitral valve repair	10 (0.90%)	10 (0.90%)	1	1	0%	0.7
Atrial septal defect occluders	34 (3.10%)	17 (1.50%)	0.016	2	1.60%	1.87
Ventricular septal defect occluders	10 (0.90%)	10 (0.90%)	1	1	0%	1.17
Paravalvular leak	10 (0.90%)	0 (0%)	0.002	-	0.90%	-
Coronary stents	54 (4.90%)	50 (4.50%)	0.688	1.08	0.40%	1.04
Covered stents	50 (4.50%)	57 (5.20%)	0.426	0.86	-0.70%	0.82
Embolic protection devices	0 (0%)	0 (0%)	1	-	0%	-

**Table 2:** The statistical comparisons between age groups for categorical clinical outcomes. For each outcome, either a chi-square test or Fisher’s exact test was used depending on cell size. The chi-square value (χ²), df, p value, and Cramér’s V (effect size) are reported. A p value <0.05 was considered statistically significant. Fisher’s exact test was applied when expected cell frequencies were <5 or when one group had zero events. Cramér’s V was calculated only for comparisons using the chi-square test and interpreted as ~0.1 (small), ~0.3 (medium), and ~0.5 (large) effect sizes CRT: cardiac resynchronization therapy; LVAD: left ventricular assist device; RVAD: right ventricular assist device; TAH: total artificial heart; IABP: intra-aortic balloon pump; ASD: atrial septal defect; VSD: ventricular septal defect; df: degrees of freedom

Outcome	Chi-square	df	p value	Cramér's V
Atrial fibrillation	4.12	1	0.042	0.043
Cardiac devices	1.95	1	0.162	0.03
Readmissions	4.85	1	0.028	0.047
Pacemakers	0.88	1	0.348	0.02
CRT	1.45	1	0.228	0.026
LVAD/RVAD/TAH	2.75	1	0.097	0.035
ASD occluders	5.14	1	0.023	0.048
Coronary stents	0.09	1	0.763	0.006
Covered stents	0.35	1	0.552	0.013
Transcatheter aortic valve replacement	0	1	1	0
Loop recorder	0	1	1	0
LVAD/RVAD/TAH	2.75	1	0.097	0.035
IABP	-	-	0.002	-
Mitral valve repair	0	1	1	0
ASD occluders	5.14	1	0.023	0.048
VSD occluders	0	1	1	0
Paravalvular leak	-	-	0.002	-
Coronary stents	0.09	1	0.763	0.006
Covered stents	0.35	1	0.552	0.013
Embolic protection devices	-	-	1	-

## Discussion

Current literature demonstrates that sex-specific and age-specific variances in CS outcomes exist. Studies have shown that women with MI-related CS present with a higher prevalence of comorbidities, often present at a more advanced age, and tend to be treated more with pharmacological management rather than procedural interventions [7,8]. However, research is limited that focuses on age-stratified, female sex-related differences in incidence, management, and outcomes of CS.

Our study highlights distinct age-related patterns in outcomes among female patients experiencing post-MI CS. Older female patients exhibited significantly higher mortality and an increased incidence of Afib, reflecting greater cardiovascular vulnerability in this population. In contrast, younger female patients, while experiencing lower mortality, showed a higher rate of emergency readmissions, pointing to the need for enhanced postdischarge care and monitoring in this group. This aligns with some research that shows higher inhospital mortality among elderly patients with CS [9] but is in stark contrast with studies that have shown that young patients (19-49 years of age) experienced higher rates of inhospital mortality [10].

Previous studies show a higher risk of complications in female patients undergoing placement of cardiac devices [11]. Notably, we found no significant differences between age groups in MACE, other arrhythmias, or complications related to cardiac device implantation. This suggests comparable risks in these specific domains across the age spectrum.

Varying treatment strategies have been explored in previous research; however, sex-specific and age-specific recommendations are absent in current guidelines [6]. Studies have shown that female patients are evaluated after significantly longer durations of symptoms, which underscores the importance of symptom recognition in female patients [6]. Women may present with delayed and atypical symptoms and are misdiagnosed, resulting in even less guideline-directed care [12].

Women often receive less frequent procedural interventions when presenting with post-MI CS [12]. Women have been shown to be less likely to receive guideline-directed medical therapies within 24 hours and at discharge, undergo diagnostic angiography, or receive mechanical circulatory support [13]. Studies have also shown that female patients are less likely to receive pulmonary artery catheterization, heart transplantation, or LVAD implantation [11]. Our findings underscore the importance of sex- and age-tailored management strategies: prioritizing mortality and arrhythmia prevention in older women, while focusing on reducing readmissions in younger patients.

We recognize several limitations of our findings. Due to the retrospective design of our study, despite our best efforts, there may be residual unaccounted confounders. PSM was performed to create balanced cohorts, allowing for a more accurate assessment of the role of age in CS outcomes. Although PSM successfully balanced the two cohorts, our study is still limited to available data within TriNetX.

## Conclusions

There are limited data about female patients with MI-related CS, particularly when examining differences between the age groups. This has made understanding characteristics, prognosis, and treatment strategies for female patients challenging. Our findings align with existing literature showing that sex- and age-related differences in CS outcomes, and specifically highlight the differences in post-MI CS among women of varying age groups.

Additional studies are needed to examine the relationship between female sex, age, and outcomes of CS. Research is also needed that explores how comorbidities impact the CS outcomes of women of varying age groups. Future research should aim to elucidate the underlying factors contributing to differences in outcomes among women of different age groups to inform more personalized care approaches for women with post-MI CS. Recognizing these differences then presents a challenge to clinicians to develop a systematic approach and early intervention for these different patient groups.
